# Assessing and Addressing Medical Residents' Knowledge Gaps in the Screening and Treatment of Metabolic Dysfunction-Associated Steatotic Liver Disease, Obesity, and Metabolic Syndrome: A Quality Improvement Project

**DOI:** 10.7759/cureus.100062

**Published:** 2025-12-25

**Authors:** Matthew Darmadi, Vani Potluri, Ryan C Thomas, Christine J Park, Olubunmi O Oladunjoye

**Affiliations:** 1 School of Medicine, Baylor College of Medicine, Houston, USA; 2 Internal Medicine, Baylor College of Medicine, Houston, USA

**Keywords:** graduate medical education, masld, metabolic syndromes, obesity, quality improvement

## Abstract

Introduction

Metabolic dysfunction-associated steatotic liver disease (MASLD), formerly known as non-alcoholic fatty liver disease (NAFLD), is the most common liver disease globally. Its rapid rise in prevalence parallels that of associated metabolic syndromes, highlighting the need for physicians well-trained in the evaluation and management of these conditions. While medical school and residency education touch on these topics, curricula often fail to address them in an adequate or longitudinal manner, leading to knowledge gaps in practice. Targeted lectures can reinforce key concepts and equip residents to manage these conditions appropriately. As such, this quality improvement study aimed to assess and enhance internal medicine residents’ confidence and understanding of MASLD, obesity, and metabolic syndrome management through a targeted educational intervention.

Methods

We integrated a structured lecture, “Obesity/Metabolic Syndrome and MASLD: The Role of the Internist,” into a Wednesday School didactic series between January 8, 2025 and February 26, 2025. Internal medicine residents attended one session each and completed pre- and post-surveys assessing confidence and knowledge in screening, risk stratification, and treatment of obesity and MASLD. Confidence was rated using four-point Likert scales, and data were visualized using stacked column charts. Participants indicated prior training through open-ended responses. Knowledge was evaluated using paired multiple-choice quizzes on the pre- and post-surveys, and scores were analyzed with paired t-tests to determine significance.

Results

Of 125 participants, 111 residents (88.8%) completed both surveys and were included in the final analysis. Confidence strongly improved across three key domains, as initially only 39.6% (n = 44), 27.9% (n = 31), and 25.2% (n = 28) of residents reported feeling “Moderately comfortable” or “Very comfortable” in managing obesity, anti-obesity medications, and MASLD, respectively. After the sessions, confidence in these realms rose to 94.6% (n = 105), 91.9% (n = 102), and 91.0% (n = 101). This increase in self-reported confidence was supported by significantly higher quiz scores after the lecture, rising from 73.9 ± 33.6 to 89.6 ± 22.5 (p < 0.001). Residents with higher baseline confidence often received prior exposure to MASLD education through conferences or clinical rotations. Qualitative feedback highlighted the relevance of the lecture and requested further instruction on pharmacologic management and institution-specific patient care pathways.

Conclusion

Targeted, lecture-based education can effectively improve internal medicine residents’ confidence and knowledge in evaluating and managing MASLD, obesity, and metabolic syndromes. Thus, integrating these interventions into residency curriculum can fill knowledge gaps and strengthen resident preparedness to address the rising burden of metabolic disease. Future directions should explore longitudinal reinforcement through multiple stages of medical training, as well as implementation in other institutions to assess clinical impact.

## Introduction

Metabolic dysfunction-associated steatotic liver disease (MASLD), previously called non-alcoholic fatty liver disease (NAFLD), is the most common liver disease in the world, affecting over 30% of all adults [1-4]. One cross-sectional epidemiological study using U.S. National Health and Nutrition Examination Survey (NHANES) data from 1999 to 2018 showed that MASLD prevalence in U.S. adults has significantly increased from 30.8% to 37.7% (p < 0.01) in the past two decades, with notable increases among women, those aged 20-45, and 61-79, and non-Hispanic White individuals [1]. Furthermore, a microsimulation model of nearly three million individuals estimated that the prevalence of MASLD in the U.S. would increase from 33.7% in 2020 to 41.4% in 2050, translating to approximately 122 million U.S. adults with MASLD in 2050 [5]. 

This recent increase in MASLD prevalence mirrors the rise of metabolic syndromes like diabetes and obesity, which are tightly associated with MASLD due to its dysfunction in lipid, cholesterol, and glucose metabolism [2-4,6]. Using systematic reviews and data provided by country collaborators and the World Health Organization, the Global Burden of Diseases (GBD) estimated that there are 529 million individuals worldwide living with diabetes, and this number is projected to increase to 1.31 billion by 2050 [7]. The GBD conducted a similar study focused on obesity and found that there are around 2.11 billion individuals with obesity worldwide, with projections reaching 3.80 billion by 2050 [8]. This sharp rise of MASLD and its associated metabolic syndromes has earned MASLD the title of a global health concern that highlights the need for physicians well-trained in the evaluation and management of these conditions [2-4]. 

Despite this glaring need, resident interns in the United States do not feel prepared to manage MASLD, obesity, and metabolic syndromes. One survey of incoming internal medicine interns at the University of Chicago found that these residents had significantly less confidence in managing liver diseases compared to pulmonary and cardiovascular conditions, with only 42% deeming their hepatology education in medical school as satisfactory [9]. U.S. residency and medical program directors and deans also agree that too little time is dedicated to hepatology education, especially in the case of metabolic dysfunction-associated steatohepatitis (MASH), a more severe form of MASLD [10]. 

The most common reasons residents feel unprepared to manage MASLD and metabolic syndromes include insufficient dedicated curricular time, lack of emphasis on practical management skills, inadequate training in lifestyle and behavioral counseling, and limited exposure to real-world clinical cases [9-12]. One way to mitigate these deficiencies is through intermittent reinforcement in the form of longitudinal education or targeted lectures. A randomized controlled trial of students from eight U.S. medical schools found that multi-modal education for weight management implemented over three years led to higher Objective Structured Clinical Examination (OSCE) scores than traditional education strategies [13], and improvements in long-term retention of nutrition knowledge were observed when education content was spread out over four weeks compared to a single lecture session [14]. Furthermore, a brief lecture-based curriculum at the University of Florida Noon Conference showed promise in improving internal medicine residents’ confidence in managing obesity and weight loss [15]. Despite the potential of these learning strategies, there is very limited evidence showing that they can improve retention of knowledge in MASLD and its associated metabolic syndromes.

The purpose of this quality improvement medical education study was to improve knowledge and comfort with the screening and management of MASLD, obesity, and metabolic syndromes in the Department of Medicine. Potential benefits resulting from this intervention can guide residency curriculum directors to improve future medical training in chronic liver diseases and metabolic syndromes, and they can lead to improved screening rates for MASLD-related liver fibrosis among high-risk patients.

## Materials and methods

Study design 

We created a lecture titled “Obesity/Metabolic Syndrome: The Role of the Internist” to be presented weekly for eight weeks at Wednesday School, a formal didactic session offered to internal medicine residents. The objectives of the lecture were to define metabolic syndrome and obesity; review the prevalence, pathophysiology, and management of obesity; identify obesity-related conditions; and focus on MASLD/NAFLD, including updated guidelines aimed at preventing progression to MASH/NASH-related fibrosis.

The lecture covered the definition of metabolic syndrome, obesity classification, medical complications of obesity, prevalence and incidence of obesity in the United States, the pathophysiology of obesity, and strategies for addressing obesity with patients. We also discussed comprehensive medical evaluation and management approaches. In addition, we reviewed the updated nomenclature and metabolic criteria for MASLD, along with its prevalence, risk factors, and disease progression. We outlined the clinical care pathway for screening for MASLD-related liver fibrosis and reviewed current and emerging pharmacologic options for the management of MASH/NASH. Two clinical cases were included to allow residents to apply the concepts discussed.

Each resident attended a single session; the weekly repetition was designed to accommodate all residents. During each session, the residents received a pre- and post-survey to assess their confidence and understanding of MASLD, obesity, and metabolic syndrome screening, risk stratification, and treatment. The surveys were designed as part of a quality improvement study, “Dissemination of Educational Material and Provider Training Metabolic Dysfunction Associated Steatotic Liver Disease (MASLD) Evaluation and Management.” The residents completed the pre-survey immediately prior to their attended lecture and the post-survey immediately after. This study was exempted from human subject review by the institutional review board.

Data collection

Complete survey contents are available in Appendix 1. To assess for confidence, the pre- and post-surveys asked the participants how comfortable they were in (1) managing obesity, (2) using anti-obesity medications, and (3) managing NAFLD/MASLD. Participants rated their confidence using a four-point Likert scale ranging from “Not at all comfortable” to “Very comfortable.” In the pre-survey, we also asked participants if they had any prior formal training in (1) weight loss management and (2) NAFLD/MASLD management using yes or no questions, with an opportunity to elaborate with a free response if “Yes” was selected. 

In addition to confidence, we evaluated understanding using paired multiple-choice quizzes included in the pre- and post-surveys. The first question asked what should be screened for in patients with NAFLD/MASLD, with the answer choices of hepatic steatosis, hepatic inflammation, hepatic fibrosis, and hepatocellular carcinoma (HCC). The second question asked how to screen for hepatic fibrosis, with the answer choices of liver ultrasound, FIB4 assessment, MRI abdomen, and CT abdomen. 

Data analysis 

Upon completion of the lecture series, we uploaded each survey result into REDCap using a matched REDCap survey for export into a spreadsheet and data analysis. We sorted confidence data into paired stacked column charts to display differences in confidence before and after the lecture, and we analyzed free responses to assess common themes in prior learning settings. In order to identify changes in scores before and after the lecture, we used a paired two-tailed t-test. We graded quizzes by assigning each question a weight of 50% and averaging the scores to obtain a mean and standard deviation. The data were analyzed using Google Sheets (Google, Inc., Mountain View, CA, USA).

## Results

A total of 125 internal medicine residents attended and participated in “Obesity/Metabolic Syndrome: The Role of the Internist.” From these participants, we excluded 14 (11.2%) survey submissions due to incompleteness, leaving 111 (88.8%) eligible surveys for analysis.

Determining changes in confidence

**Figure 1:**
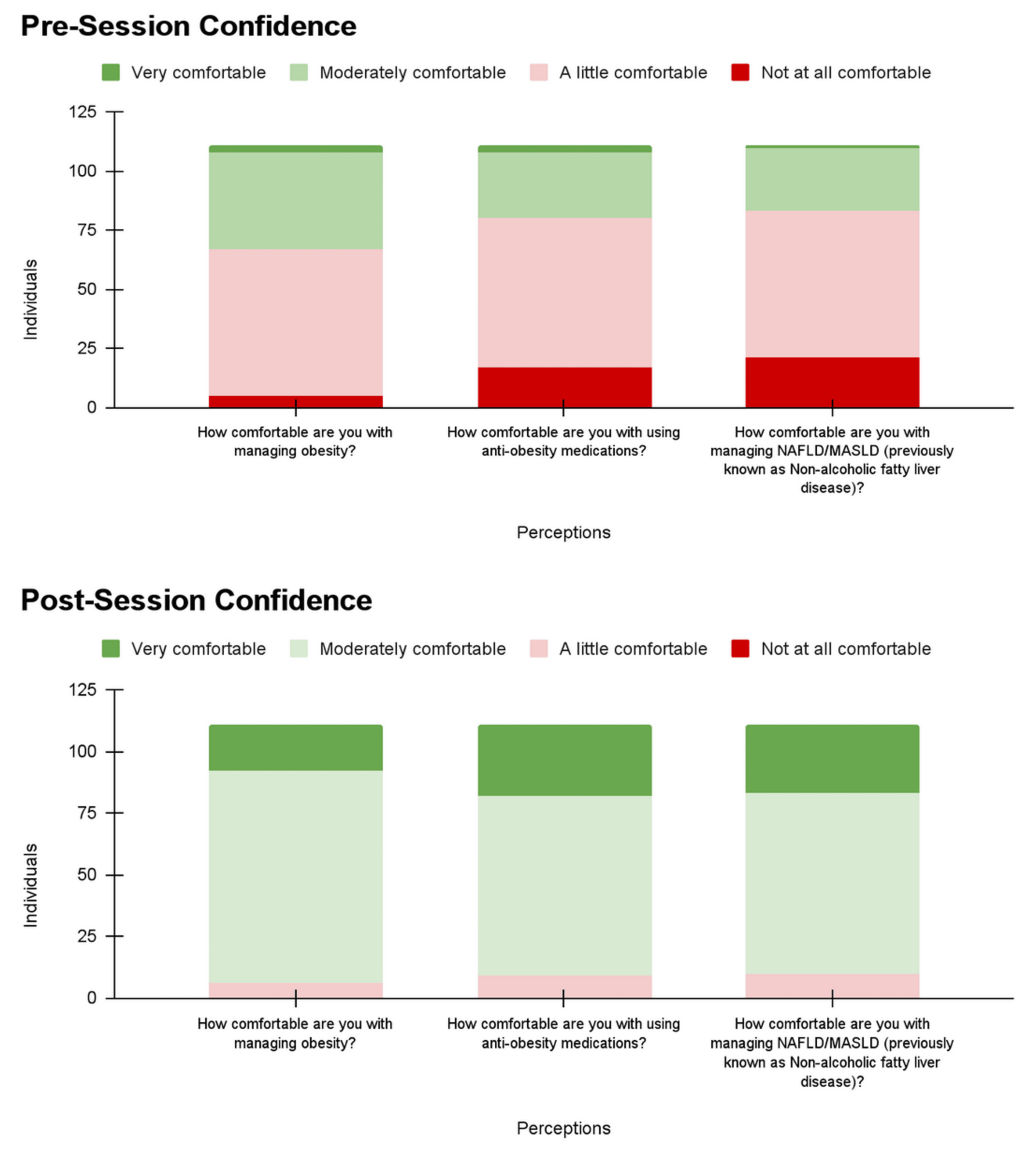
Confidence of internal medicine residents regarding metabolic syndrome and MASLD management before and after “Obesity/Metabolic Syndrome: The Role of the Internist,” January 2025 - February 2025. Confidence was measured using a four-point Likert scale ranging from “Not at all comfortable” to “Very comfortable.” NAFLD, non-alcoholic fatty liver disease; MASLD, metabolic dysfunction-associated steatotic liver disease

The four-point Likert scales demonstrated an overall increase in confidence regarding the management of metabolic syndromes and MASLD. Before the lecture, only 44 (39.6%) residents felt “Moderately comfortable” or “Very comfortable” in managing obesity, compared with 105 (94.6%) residents after the lecture (Figure 1). Similar improvements were observed in confidence with managing anti-obesity medications and managing NAFLD/MASLD, increasing from 31 (27.9%) to 102 (91.9%) and 28 (25.2%) to 101 (91.0%), respectively (Figure 1). Notably, after the lecture, no residents reported feeling “Not at all comfortable” managing obesity, anti-obesity medications, and NAFLD/MASLD (Figure 1).

Prior training 

**Figure 2:**
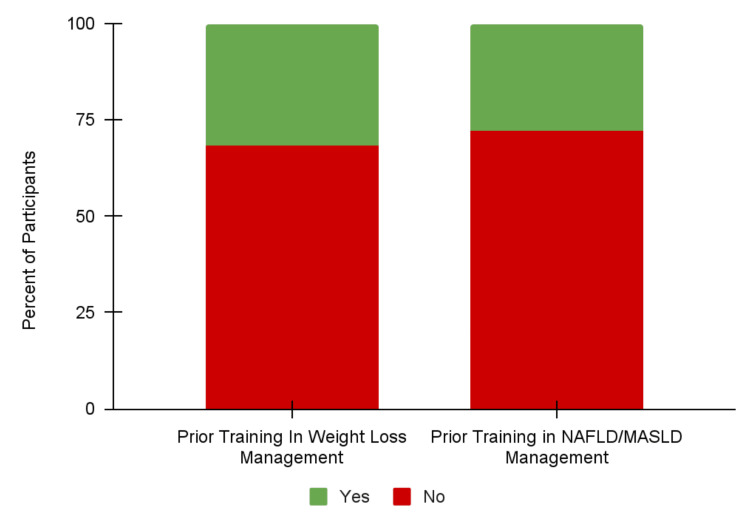
Percent of residents who had training in weight loss management and/or NAFLD/MASLD management prior to “Obesity/Metabolic Syndrome: The Role of the Internist,” January 2025 - February 2025. NAFLD, non-alcoholic fatty liver disease; MASLD, metabolic dysfunction-associated steatotic liver disease

Of the residents who reported higher confidence before the lecture, most had previously learned about metabolic syndromes, obesity, and MASLD management from a conference/lecture or a rotation focusing on these conditions. Specifically, 35 (31.5%) of the residents had prior training in weight loss management, and 31 (27.9%) of the residents had prior training in NAFLD/MASLD management (Figure 2). The most commonly cited conference/lecture experiences were a part of the Internal Medicine residency curriculum, including Noon Conference sessions featuring high-yield instruction once per week and Wednesday School didactics every other block. Many residents with prior training in weight loss management gained experience in specialty clinics, and many with training in NAFLD/MASLD learned from gastroenterology and hepatology rotations. Only three (2.7%) and four (3.6%) residents had prior training in weight loss management and NAFLD/MASLD management, respectively, from medical school curricula.

Knowledge assessment

**Figure 3:**
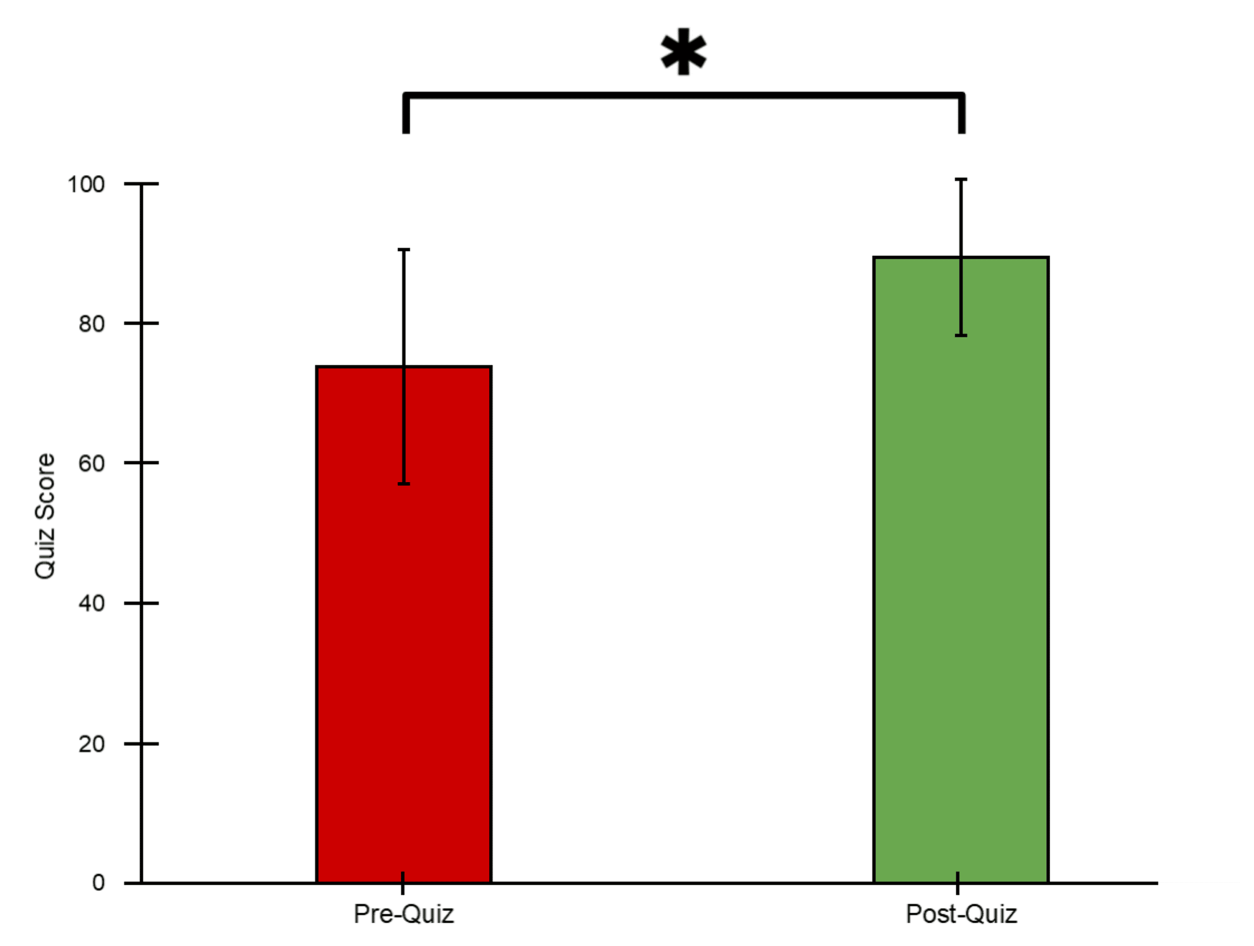
Pre- and post-survey quiz scores of internal medicine residents attending “Obesity/Metabolic Syndrome: The Role of the Internist,” January 2025 - February 2025. Error bars indicate respective standard deviations. An asterisk indicates p < 0.001.

In addition to the confidence assessment, the residents took identical, paired quizzes before and after the lecture to evaluate the knowledge gained from the event. Before the lecture, the residents scored an average of 73.9 ± 33.6 (Figure 3). After the lecture, the residents scored an average of 89.6 ± 22.5 (Figure 3). The paired, two-tailed t-test revealed that the post-quiz scores were significantly higher, with a p < 0.001.

## Discussion

Despite the marked need for physicians trained in MASLD and its associated metabolic syndromes, residents do not feel prepared to address these conditions in practice due to a lack of focused education and real-world experience during their training [9-12]. This project aimed to target this gap in confidence and knowledge by implementing “Obesity/Metabolic Syndrome: The Role of the Internist” into formal didactic sessions offered to internal medicine residents. We found that this intervention not only increased residents’ confidence in managing these conditions but also significantly improved their understanding, as shown through paired pre- and post-quizzes. In this section, we reflect on the residents’ thoughts on the lecture and comparisons to another cohort. We also highlight implications of this study and address limitations and future directions. 

Resident perceptions 

At the end of the post-survey, we included one optional question asking for “anything else we could have improved or focused on,” which was answered by 39 (35.1%) residents. Of these residents, 23 (59.0%) mentioned that the lecture was “great” or a similar equivalent. One resident stated, “LOVED this presentation! Thank you so much!” The remaining 16 comments (41%) focused on areas for improvement or further elaboration, with the main themes asking about pharmaceutical management and location-specific procedures. Five comments (12.8%) requested more information about specific medications and their effects. For example, one resident wanted more details on “dosing titration of these medicines, length of (treatment), and when to stop,” and another suggested “more case based learning, perhaps to facilitate understanding weight loss meds/common side effects.” Five different comments (12.8%) asked about specific workplace procedures to improve local patient care. One resident requested “specific resources available to us” to serve primarily uninsured populations. Another resident asked, “how to get GLP-1 agonists for our patients,” specifically in local pavilions. These comments show that the residents welcome “Obesity/Metabolic Syndrome: The Role of the Internist” as a part of their curriculum and desire further information on pharmaceutical treatment and local institutional procedures.

Cohort comparisons and beyond 

The need for more MASLD and metabolic syndrome education extends beyond U.S. residency programs. Primary Care and Family Medicine residents across Canada were surveyed in 2023, and only 11% of the residents felt that they had adequate exposure to MASLD [16]. Furthermore, only 35% demonstrated a reasonable level of knowledge when quizzed on the survey, and 94% recommended additional instruction focusing on MASLD [16]. This lack of MASLD preparedness is also seen on a global scale. One study published in 2022 examined the preparedness of 102 countries to address the rise of MASLD and found that every single country lacked an adequate, comprehensive health response for MASLD [17]. Notably, 32 countries scored a 0 out of 100 in preparedness [17], further highlighting the need for improved MASLD education. 

Implications 

Our findings suggest that the implementation of targeted lectures can successfully improve residents’ confidence and understanding of MASLD, obesity, and metabolic syndrome management. These benefits align strongly with a recent study that reported improvements in confidence and knowledge from both a traditional slide-based MASLD lecture and a gamified version [18]. Therefore, other residency programs would also benefit from implementing a targeted lecture on MASLD as a crucial form of multi-modal education approach [13-15].

Limitations

While this study demonstrates the benefits of targeted educational interventions for MASLD, obesity, and metabolic syndromes, there are several limitations that should be addressed. First, this study did not assess retention of knowledge at multiple time points after the lecture, so we were only able to show that the intervention improved short-term confidence and understanding. Due to the short time frame of the study, we were also unable to determine if increased understanding resulting from the intervention translated into improved patient care or outcomes. Social desirability bias in self-reported confidence is also a potential limitation, as there is a possibility that some may have overstated their confidence or abilities to appear better or more competent than they actually are. In addition, the simplicity of the knowledge quiz questions could also have limited the sensitivity of detecting differences in knowledge. Lastly, we did not test the lecture series at other institutions or residency programs, which could have revealed cohort-specific differences in our analyses.

## Conclusions

This study underscores the value of targeted educational interventions focused on MASLD, obesity, and metabolic syndrome evaluation and management. We found that these lectures not only enhance learners’ confidence but also strengthen understanding, highlighting their power to improve both education and patient care. Residency programs should implement similar targeted lectures or elective courses into their curricula to reinforce residents’ preparedness in managing MASLD and metabolic syndrome care.

Future directions should aim to address limitations by increasing the breadth of the targeted lectures to other residency programs and following the residents and their patients’ outcomes for a longer period of time. This strategy would also show the long-term effects of the lecture series on resident knowledge retention and patient outcomes. Additional directions include the implementation of similar lectures throughout the medical school curriculum. Examining these medical students’ confidence and understanding of MASLD, obesity, and metabolic syndromes during their residencies would further inform the long-term effects of the lectures on understanding and knowledge retention.

## Appendices

Appendix 1

**Table 1:** Pre- and post-survey questions. NAFLD, non-alcoholic fatty liver disease; MASLD, metabolic dysfunction-associated steatotic liver disease

Pre- and Post-Survey Questions			
Pre-Activity Questions			
1. How comfortable are you with managing obesity?			
a. Not at all comfortable	b. A little comfortable	c. Moderately comfortable	d. Very comfortable
2. How comfortable are you with using anti-obesity medications?			
a. Not at all comfortable	b. A little comfortable	c. Moderately comfortable	d. Very comfortable
3. How comfortable are you with managing NAFLD/MASLD (previously known as Non-alcoholic fatty liver disease)?			
a. Not at all comfortable	b. A little comfortable	c. Moderately comfortable	d. Very comfortable
4. Have you had any formal teaching in weight loss management?			
a. No	b. Yes	If yes, please write where:	
5. Have you had any formal teaching in NAFLD/MASLD management?			
a. No	b. Yes	If yes, please write where:	
6. What do we screen for in patients with NAFLD/MASLD?			
a. Hepatic steatosis	b. Hepatic inflammation	c. Hepatic fibrosis	d. Hepatocellular carcinoma (HCC)
7. How do we screen for hepatic fibrosis?			
a. Liver ultrasound	b. FIB4 assessment	c. MRI abdomen	d. CT abdomen
Post-Activity Questions			
1. After this lecture, how comfortable are you with managing obesity?			
a. Not at all comfortable	b. A little comfortable	c. Moderately comfortable	d. Very comfortable
2. After this lecture, how comfortable are you with using anti-obesity medications?			
a. Not at all comfortable	b. A little comfortable	c. Moderately comfortable	d. Very comfortable
3. After this lecture, how comfortable are you with managing NAFLD/MASLD (previously known as Non-alcoholic fatty liver disease)?			
a. Not at all comfortable	b. A little comfortable	c. Moderately comfortable	d. Very comfortable
4. What do we screen for in patients with NAFLD/MASLD?			
a. Hepatic steatosis	b. Hepatic inflammation	c. Hepatic fibrosis	d. Hepatocellular carcinoma (HCC)
5. How do we screen for hepatic fibrosis?			
a. Liver ultrasound	b. FIB4 assessment	c. MRI abdomen	d. CT abdomen
6. Anything else we could have improved or focused on:			
